# Fluorescent Nanoporous Gene Drugs with Fenton-like Catalysis Vector Research

**DOI:** 10.3390/nano16020120

**Published:** 2026-01-16

**Authors:** Yulin Li, Jianjun Pan, Lili Xu, Yan Sun, Tong Li

**Affiliations:** 1 The Key Laboratory for Crop Production and Smart Agriculture of Yunnan Province, Yunnan Agricultural University, Kunming 650201, China; yulinli812@163.com; 2 College of Life Science and Biotechnology, Heilongjiang Bayi Agriculture University, Daqing 163319, China; jianjunpan815@163.com; 3 School of Data Science and Engineering, Kunming City College, Kunming 650106, China; xll8911623@163.com; 4 College of Big Data, Yunnan Agricultural University, Kunming 650201, China

**Keywords:** liver cancer, nanogene drug carrier, Fenton-like reaction, siRNA, collaborative therapy

## Abstract

A multifunctional diagnosis and treatment carrier, ZIF-8@CDs, based on carbon quantum dots (CDs) and the zeolitic imidazolate framework-8 (ZIF-8) metal–organic framework which serves as a core structure for constructing the responsive delivery platform, is developed in this paper. The anticancer drug doxorubicin (DOX) and Survivin oligo (siRNA) are loaded to form a ZIF-8@CDs/DOX@siRNA dual loading platform. CDs of 5–10 nm are synthesized by the solvent method and combined with ZIF-8. Electron microscopy shows that the composites are nearly spherical particles of approximately 200 nm, and the surface potential decreases from +36 mV before loading CDs to +25.7 mV after loading. The composite system shows unique advantages: (1) It has Fenton-like catalytic activity, catalyzes H_2_O_2_ to generate hydroxyl radicals, and consumes glutathione in the tumor microenvironment. The level of reactive oxygen species (ROS) in the ZIF-8@CDs group is significantly higher than that in the control group. (2) To achieve visual diagnosis and treatment, its fluorescence intensity is superior to that of the traditional Fluorescein isothiocyanate (FITC)-labeled vector; (3) It has a high loading capacity, with the loading amount of small nucleic acids reaching 36.25 μg/mg, and the uptake rate of siRNA by liver cancer cells is relatively ideal. The ZIF-8@CDs/DOX@siRNA dual-loading system is further constructed. Flow cytometry shows that the apoptosis rate of HepG2 cells induced by the ZIF-8@CDs/DOX@siRNA dual-loading system is 49%, which is significantly higher than that of the single-loading system (ZIF-8@CDs/DOX: 34.3%, ZIF-8@CDs@siRNA: 24.2%) and the blank vector (ZIF-8@CDs: 12.6%). The platform provides a new strategy for the integration of tumor diagnosis and treatment through the multi-mechanism synergy of chemical kinetic therapy, gene silencing and chemotherapy.

## 1. Introduction

With the development of society, the incidence of liver cancer has been rising year by year, becoming one of the most severe public health issues globally and posing a serious threat to human health [1]. However, the treatment of liver cancer still faces many challenges, including strong side effects of treatment methods, the tumor’s tendency to develop drug resistance, and significant tumor heterogeneity. Therefore, there is an urgent need to develop efficient and low-toxicity treatment strategies—among these, targeted therapy has become a research hotspot [2] because it can precisely locate and kill tumor cells.

In recent years, multifunctional nano-drug delivery systems have shown great potential in tumor-targeted therapy. Among them, metal–organic frameworks (MOFs), with their tunable porous structures, high specific surface area, and good biocompatibility, have provided a new direction for cancer combination therapy [3,4,5,6,7,8,9,10,11,12,13]. As a typical member of the MOF family, ZIF-8 has become an ideal candidate material for nanodrug delivery due to its excellent chemical stability, controllable pore size, and tunable surface properties. However, most existing ZIF-8-based delivery systems are largely limited to single-modal therapy, which not only hinders therapeutic efficacy but also restricts the potential for clinical translation.

To address the above issues, in this paper, a CDs [14,15,16,17,18,19,20] modified ZIF-8 composite carrier ZIF-8@CDs [21] is constructed. The chemotherapy-gene synergistic therapy is achieved by co-loading DOX and siRNA, and its diagnosis and treatment integration function is innovatively developed [22,23].

In this paper, we develop a stable method for combining CDs with ZIF-8 [24,25,26] based on existing research and confirm its successful construction through Transmission Electron Microscope/Scanning Electron Microscopy (TEM/SEM) and other characterization techniques. The key breakthrough lies in the discovery of ZIF-8@CDs with significant Fenton-like catalytic activity [27,28,29,30,31]: TMB chromogenic and glutathione (GSH) consumption experiments confirm that ZIF-8@CDs catalyze H_2_O_2_ to generate hydroxyl radicals (· OH) and consume GSH in the tumor microenvironment. Intracellular ROS surge and effectively induce tumor cell apoptosis [32,33,34,35]. This provides some molecular mechanism evidence for chemodynamic synergistic therapy.

In terms of functional verification, drug loading experiments show that the system can simultaneously load DOX inside the channel and siRNA on the surface of the carrier to form a dual-drug synergistic delivery system [36,37,38,39]. Hepatocellular carcinoma cell HepG2 experiments confirm: (1) the carrier can be efficiently internalized; (2) compared with single-drug treatment, the composite carrier significantly increases the apoptosis rate through a synergistic effect; (3) CDs endow the system with real-time fluorescence tracing ability, which enables the integration of diagnosis and treatment [16,18,19].

Therefore, this paper aims to construct a CDs-modified ZIF-8 composite carrier co-delivering DOX and siRNA, and to evaluate its synergistic therapeutic effects on liver cancer cells. In addition, the paper clarifies the synergistic mechanism of chemo-gene-chemokinetics therapy by examining intracellular ROS levels and GSH depletion. This integrated approach, combining multifunctional carrier construction with mechanism elucidation, provides a potential new strategy for precise diagnosis and efficient treatment of liver cancer.

## 2. Materials and Methods

### 2.1. Materials

Zinc nitrate hexahydrate (99%) was purchased from Aladdin Biochemical Technology Co., Ltd. in Shanghai, China. 2-Methylimidazole (99%) was purchased from Aladdin Biochemical Technology Co., Ltd. Sucrose was purchased from Tianjin Damao Reagent Co., Ltd. in Tianjin, China. Concentrated sulfuric acid (65%), glucose, methanol and anhydrous ethanol were purchased from Tianjin Yongda Chemical Reagent Co., Ltd. in Tianjin, China. Dimethyl sulfoxide (DMSO) was purchased from Shanghai Dibai Biotechnology Co., Ltd. in Shanghai, China. FITC was purchased from Thermo Fisher Scientific  in Waltham, MA, USA. DMEM (high glucose) medium was purchased from Hangzhou Sijiqing Company in Hangzhou, Zhejiang Province, China. 4% paraformaldehyde solution, trypsin cell digestive juice, 1% penicillin and streptomycin were purchased from Biosharp Biotechnology Co., Ltd. in Hefei, Anhui Province, China. Fetal bovine serum (FBS) was purchased from Beijing Solarbio Science&Technology Co., Ltd. in Beijing, China. Annexin V-APC apoptosis detection kit was purchased from Wuhan Punosai Life Science and Technology Co., Ltd. in Wuhan, Hubei Province, China. Anti-fluorescence quencher was purchased from Beijing Zhongshan Jinqiao Biotechnology Co., Ltd. in Beijing, China. Diethyl pyrocarbonate (DEPC) water was Milli-Q pure water treated with DEPC and sterilized under high temperature and high pressure. All solutions containing siRNA were prepared using water treated with RNase-free DEPC. All experiments used ultrapure deionized water.

Synthetically prepared apoptosis-inducing siRNA: a 20 bp short-chain herring sperm DNA; siRNA is composed of the following sequences: Sense 5′-GGCUGUUCCUGAAAUAATT-3′ and Antisense 5′-UUAUUUCUCAGGAACAGCCTT-3′. The sequence of negative control siRNA is as follows: Sense 5′-UUCUCCGAACGUGUCACGUTT-3′ and Antisense 5′-ACGUGACACGUUCGGAGAATT-3′. All siRNAs were purchased from Shanghai Sangon Biotech Co., Ltd. in Shanghai, China.

### 2.2. Methods

#### 2.2.1. Preparation of CDs

Preparation of sucrose solution: Sucrose (0.172 g) was dissolved in 50 mL of methanol. Preparation of sulfuric acid solution: concentrated sulfuric acid (0.01 mL) was dissolved in 100 mL methanol, and the mixture was sonicated until it was completely dissolved. Concentrated sulfuric acid (0.005 mL) was added to the sucrose solution and ultrasonically dissolved. The mixture was heated at 180 °C for 6 h, dialyzed after natural cooling to obtain green carbon quantum dots (G-CDs). For convenient storage, it was freeze-dried to obtain powdered G-CDs; Neutral red (0.1 g) and thiourea (0.1 g) were dissolved in pure water (20 mL), transferred to a stainless steel autoclave, heated at 180 °C for 6 h, naturally cooled to room temperature, and dialyzed with ultrapure water using a dialysis bag (100 Da) for 24 h to obtain red carbon quantum dots (R-CDs). To verify the fluorescence intensity and stability of R-CDs and G-CDs, both solutions were subjected to a 48 h storage test.

#### 2.2.2. Preparation of ZIF-8@CDs

Solution A: Zinc nitrate hexahydrate (0.3 g) was dissolved in methanol (11.3 g); Solution B: 2-methylimidazole (0.66 g) was dissolved in methanol (11.0 g). The mixture of A and B solutions were placed on a magnetic stirrer and stirred violently for 5 min until the color changed from colorless to milky white. Afterwards, the mixture was transferred into a stainless steel reactor lined with polytetrafluoroethylene and heated at 150 °C for 5 h. The resulting precipitate was centrifuged at 8000 rpm for 10 min and washed three times with methanol and anhydrous ethanol, respectively. Finally, the centrifuged product was suspended in ethanol and dried under vacuum overnight to obtain ZIF-8 nanoparticles. The preparation principle of ZIF-8@CDs nanoparticles was the same as above, but the powdered CDs needed to be fully dissolved in methanol in advance.

#### 2.2.3. Modification of FITC Fluorescein

In order to facilitate a more intuitive comparison of various characteristics of ZIF-8@CDs, such as stability, strength, cellular uptake efficiency, the distribution of carbon quantum dots after entering cells, and whether various characteristics can function properly for visual monitoring, fluorescent labeling was used to stain and modify pure ZIF-8 nanoparticles, making it easier to serve as a control comparison with ZIF-8@CDs in cellular uptake experiments. Simply put, it involved placing the pretreated ZIF-8 into a sterile centrifuge tube (1.5 mL), adding sterile deionized water (1 mL) to resuspend pretreated ZIF-8 nanoparticles, and sonicating until the mixture was uniform. The mixture was centrifuged (10,000 rpm, 5 min) with an ultracentrifuge to remove the supernatant. After removing the supernatant, FITC (50 mL) was added to the centrifuge tube and adsorbed overnight under dark conditions. After the adsorption time, the excess dye was washed with sterile deionized water to obtain ZIF-8@FITC, which was resuspended in serum-free medium for use.

#### 2.2.4. Preparation of ZIF-8@CDs/DOX@siRNA

An appropriate amount of ZIF-8@CDs and DOX (at a mass ratio of 2:1) was taken, the two components were mixed, deionized water (1 mL) was added, and the resulting mixture was subjected to ultrasonic treatment for 20 min. Such treatment enabled the uniform loading of DOX on the surface of ZIF-8@CDs and internal pores. The mixture was usually left overnight to fully realize the loading of doxorubicin and was then centrifuged twice at 8000 rpm for 10 min using a high-speed centrifuge. Centrifugation of the mixture served to remove unadsorbed or insufficiently adsorbed DOX and other impurities; the resulting precipitate was then dried overnight under vacuum (80 °C) to obtain the DOX-loaded composite nano-drug carrier ZIF-8@CDs/DOX. After the siRNA (1 OD) was centrifuged for 10 min, RNase-free DEPC water (37.5 μL) was added. Then the mixture was mixed thoroughly with ZIF-8@CDs/DOX by vortexing to obtain ZIF-8@CDs/DOX@siRNA (prepared fresh for immediate use). Maintain an RNase-free environment throughout to prevent siRNA degradation, and perform all operations in the dark to prevent quenching of CDs fluorescence and siRNA inactivation. ZIF-8@CDs/DOX nanoparticles were incubated with the diluted siRNA under dark conditions for 6 h, washed twice with DEPC water, and centrifuged to remove impurities, and finally the resulting ZIF-8@CDs/DOX@siRNA was added to serum-free medium (1 mL) for resuspension.

#### 2.2.5. Characterization

After preparing the powder sample, the morphology of the nanoparticles was observed by a Zeiss SIGMA field emission SEM(Zeiss AG in Oberkochen, Baden-Württemberg, Germany). The morphology and structure of monodisperse ZIF-8, monodisperse CDs, and monodisperse ZIF-8@CDs were photographed by TEM (JEOL TEM-2100 Plus in Tokyo, Japan) at 120 kV. The monodisperse ZIF-8 and ZIF-8@CDs were characterized using a Rigaku D/max-2200 X-ray diffractometer (XRD, Rigaku Corporation Tokyo, Japan) to confirm their crystalline state. The diffractometer operated at 40 kV and 40 mA with Cu Kα radiation, and the 2θ scanning range was set to 5–40°. Fourier Transform Infrared Spectroscopy (FTIR) spectra were collected by Thermo, Nicolet380 (Thermo Fisher Scientific in Waltham, MA, USA). The sample powder was mixed with potassium bromide (KBr) at a ratio of 1:40, ground into a fine powder, pressed into pellets, and then tested using the FTIR spectrometer. The material to be tested was resuspended in a suitable solution, and its zeta potential and particle size were analyzed and recorded using the ZEN3700 laser particle size analyzer (Malvern Panalytical in Malvern, Worcestershire, UK). The samples were analyzed using a Micromeritics ASAP 2460 specific surface area analyzer (Micromeritics Instrument Corporation in Norcross, GA, USA). The BET diagram, along with the slope and intercept of the BET isotherm, was used to calculate the pore size distribution and specific surface area of ZIF-8 and ZIF-8@CDs. The ultraviolet-visible (UV-Vis) absorption spectra of the samples were measured using a Jenway 6715 UV-Vis spectrophotometer (Jenway Group, Bibby Scientific in Stone, Staffordshire, UK). Before testing, the samples were resuspended in a cuvette, and the scan range was set from 200 to 700 nm. The fluorescence emission spectra and fluorescence intensity of the samples were measured using a F-4500 Fluorescence Spectrophotometer (FS) (Hitachi High-Technologies Corporation in Tokyo, Japan). Before testing, the samples were resuspended in an appropriate solution, with the excitation wavelength set to 420 nm and the emission scan range set from 300 to 600 nm. The uptake of materials by cells was measured at 488 nm (green) and 633 nm (red) using a TCS SP8 Confocal laser scanning microscopy (CLSM) (Leica Microsystems GmbH in Wetzlar, Baden-Württemberg, Germany). Before testing, the coverslips with the mixed cells and materials were placed upside down on slides containing DAPI anti-fade mounting medium, and each slide was properly labeled.

#### 2.2.6. Detection of Fenton-like Catalytic Activity of ZIF-8@CDs

It is based on the oxidation of 3,3′,5,5′ -tetramethylbenzidine (TMB) to ox-TMB in the presence of H_2_O_2_. With TMB as the probe, the Fenton-like catalytic activity of ZIF-8@CDs was evaluated. Typically, 100 μL of ZIF-8@CDs dispersion (1 mg/mL) and 60 μL of TMB solution (10 mM) were added to 0.2 M buffer (pH = 4.0). The mixture was then incubated at 50 °C for 30 min in a total volume of 2 mL. The UV-Vis absorption spectrum and the absorbance at 652 nm were recorded, and the resulting data were collected and analyzed. The GSH detection system was established simultaneously, and ox-TMB was reduced to a colorless form by GSH. The specific operation is as follows: 1 mg/mL ZIF-8@CDs (100 μL) and 10 mM TMB (60 μL) were supplemented into 40 μL of pH 4.0 buffer. The experimental group was additionally designed to include 60 μL of 50 mM GSH or 60 μL of 100 mM GSH. The mixture (final volume 2 mL) was then incubated at 50 °C for 30 min. UV-Vis absorption spectra and the absorbance at 652 nm were recorded, and the resulting data were collected and analyzed.

#### 2.2.7. Study on the Adsorption of DNA by ZIF-8@CDs

Before ZIF-8@CDs adsorbed siRNA, a 20 bp fragment of herring sperm DNA was selected for the adsorption experiment with ZIF-8@CDs. Firstly, 5 equal aliquots of ZIF-8@CDs were weighed out, taken, and placed into centrifuge tubes marked with appropriate labels. After adding DEPC water and mixing thoroughly, 200 μL of pre-prepared herring sperm DNA (concentration gradient: 100, 80, 60, 30, 10 ng/mL) was added to each tube, gently shaken, and then allowed to stand undisturbed for 6 h to ensure full contact and adsorption. After adsorption, the treated DNA samples were subjected to agarose gel electrophoresis to evaluate the adsorption capacity of the ZIF-8@CDs for DNA fragments. Finally, after adsorption, the supernatants were collected from all centrifuge tubes, and the DNA concentration of each supernatant was detected using a Nanodrop 2000 microspectrophotometer (Thermo Fisher Scientific in Wilmington, DE, USA).

#### 2.2.8. Assessment of Cellular Uptake

Logarithmic phase HepG2 cells were seeded at 4 × 10^4^ cells per well in 24-well plates containing coverslips and cultured at 37 °C with 5% CO_2_ for 24 h. The medium was then discarded and replaced with serum-free medium containing the materials (ZIF-8@FITC, ZIF-8@CDs, and ZIF-8@CDs@siRNA, at a working concentration of 128 μg/mL). After 4 h, the cells were washed with phosphate-buffered saline (PBS) and then replaced with complete medium for 24 h. Cells were fixed with 4% paraformaldehyde (PFA) for 20 min, washed, and treated with 0.1% Triton X-100 for 20 min to permeabilize the cells, followed by three thorough washes with PBS. The coverslips were then mounted on glass slides with DAPI-containing antifade reagent and observed under a confocal laser scanning microscope to assess cellular uptake of the materials.

#### 2.2.9. Detection of Intracellular ROS

The intracellular ROS level was detected by the 2′, 7′-dichlorofluorescein (DCFH-DA) probe. HepG2 cells were inoculated into 24-well plates at a seeding density of 4 × 10^4^ cells per well, followed by incubation at 37 °C in a 5% CO_2_ atmosphere for 24 h, the complete medium was discarded, and 128 μg/mL of ZIF-8, ZIF-8@CDs, ZIF-8@CDs/DOX, ZIF-8@CDs@siRNA, and ZIF-8@CDs/DOX@siRNA were deposited into the wells and repeated three times. The fluorescence intensity was detected by a microplate reader or laser confocal microscopy. After 4 h, the medium containing nanoparticles was removed from the wells, 200 μL of DCFH-DA (0.1 M) was added to each well, and the mixture was incubated at 37 °C for 20 min. After incubation, the excess probe solution was removed and the cells were washed three times with DMEM. To observe and compare the changes in reactive oxygen species within cells, fluorescence images were taken at various time points or within different treatment groups.

#### 2.2.10. Cytotoxicity Detection

In order to verify the low toxicity of nanomaterials, HepG2 cells were seeded in 96-well cell culture plates, cultured in 150 μL complete medium for 12 h at 37 °C and 5% CO_2_; When the cells grew to about 80%, ZIF-8, ZIF-8@FITC, ZIF-8@CDs, ZIF-8@CDs/DOX, ZIF-8@CDs@siRNA, and ZIF-8@CDs/DOX@siRNA were added at concentrations of 1, 2, 4, 8, 16, 32, 64, and 128 μg/mL for 4 h. Then the cells were placed in complete culture medium for 12 h to study the effect of each material on cell viability. After removing the mixture with an oil pump, 20 μL of 3-(4,5-dimethyl-2-thiazolyl)-2,5-diphenyltetrazolium bromide (MTT) solution (5 mg/mL) was administered to each well and incubated at 37 °C for 4 h. The medium was first discarded, and DMSO (100 μL) was then administered to each well. The plate was subsequently placed in the dark for 15 min until the purple crystals were completely dissolved. The absorbance at 490 nm was measured by a microplate reader. Cell viability was calculated according to OD value.

#### 2.2.11. Cell Apoptosis Assay

In order to evaluate the induction effect of the nanocomposite carrier on tumor cell apoptosis, siRNA targeting the Survivin gene was selected for carrier delivery in this experiment. Survivin is one of the smallest apoptosis inhibitory proteins that are highly expressed in tumor cells. An siRNA oligo with a length of 21 bp was designed and synthesized for subsequent experiments.

Logarithmically growing HepG2 cells were picked and incubated in various culture media (ZIF-8, ZIF-8@FITC, ZIF-8@CDs, ZIF-8@CDs/DOX, ZIF-8@CDs@siRNA, and ZIF-8@CDs/DOX@siRNA) at a uniform final concentration of 128 μg/mL. After 24 h of culture, the cells were collected into enzyme-free sterile centrifuge tubes, washed twice with PBS, and then treated with EDTA-free trypsin digestion solution. The suspension was collected, centrifuged, and resuspended for cell counting. After centrifugation under the same conditions, approximately 2 × 10^5^ cells from each group were resuspended in 195 μL of Annexin V-APC binding buffer. Subsequently, 5 μL of Annexin V-APC and 10 μL of propidium iodide (PI) staining solution were added. The mixture was gently vortexed and incubated for 10–20 min at room temperature in the dark. After the reaction was completed, the samples were placed in an ice bath and stored in the dark, and flow cytometry was used for detection as soon as possible.

## 3. Results

**Scheme 1:**
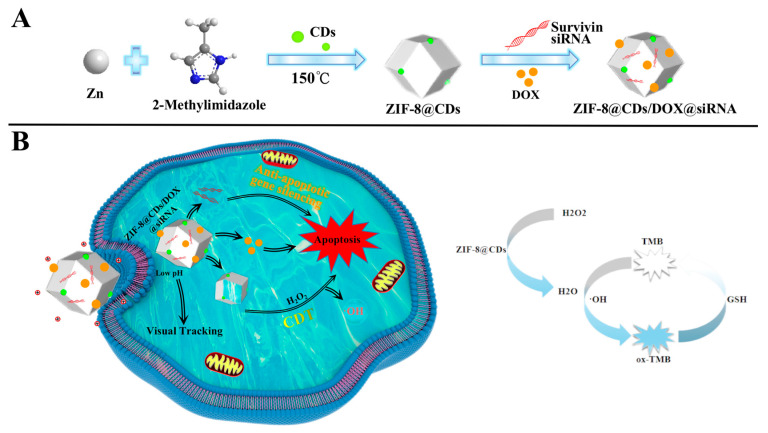
(**A**) Schematic diagram of the preparation of ZIF-8@CDs/DOX@siRNA; (**B**) Schematic diagram of the cancer therapy cascade process of ZIF-8@CDs/DOX@siRNA.

In this paper, a multifunctional nano-gene drug carrier ZIF-8@CDs was successfully constructed (Scheme 1A). Its core design realizes the simultaneous and efficient loading of chemotherapeutic drug doxorubicin and siRNA by regulating the pore structure, which solves the problem of drug inactivation and cell membrane penetration pain points in traditional delivery [40,41,42]. CDs modification endows the carrier with visual traceability, enabling real-time monitoring of drug uptake and intracellular distribution (Scheme 1A). The key finding is that ZIF-8@CDs exhibit Fenton-like catalytic activity in the tumor microenvironment. Experiments have confirmed that it can catalyze the production of a large amount of ·OH and consume GSH, resulting in a surge in ROS levels (Scheme 1B). It synergistically promotes tumor cell apoptosis by inducing oxidative stress damage and destroying antioxidant defense systems [34,43,44]. At the same time, the siRNA stably carried by the carrier can silence tumor drug-resistant genes [45,46], and the combination of siRNA with adriamycin can significantly enhance efficacy [47,48], overcome chemotherapy resistance, and achieve synergistic treatment [49].

### 3.1. Morphological Physicochemical Characterization of CDs

**Figure 1:**
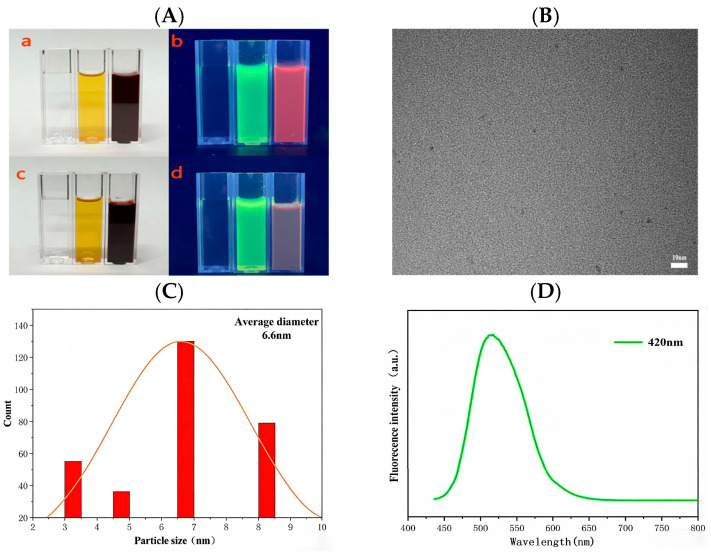
(**A**) (**a**). From left to right: distilled water, G-CDs, and R-CDs under white light; (**b**). From left to right: distilled water, G-CDs, and R-CDs under 365 nm fluorescent excitation; (**c**). Distilled water, G-CDs, and R-CDs photographed under white light after 48 h; (**d**). Distilled water, G-CDs, and R-CDs under 365 nm fluorescent excitation after 48 h; (**B**) TEM images of CDs. Scale bar: 10 nm; (**C**) The size distribution of CDs obtained by TEM image analysis; (**D**) FS of the as-synthesized CDs. The spectrum was recorded with an excitation wavelength of 420 nm. The CDs exhibit a strong green photoluminescence with a maximum emission intensity at 525 nm, resulting in a large Stokes Shift of approximately 105 nm.

The fluorescence intensity of CDs prepared in methanol medium was the highest, and the optical stability of the G-CDs and R-CDs fluorescent products was tested via a 48-h storage experiment. The results (Figure 1A) showed that G-CDs still maintained stable fluorescence properties after 48 h of storage, while the fluorescence intensity of R-CDs decreased significantly, indicating that there may be some defects in their structural stability or anti-quenching ability. Based on the comprehensive advantages of G-CDs in preparative purity and optical stability, it was selected as the subsequent research subject and was designated as CDs in subsequent experiments.

The morphological and structural properties of CDs were characterized by TEM (Figure 1B). CDs were uniform spherical particles with a size distribution of 5–10 nm and good monodispersity. It was confirmed that the synthesis method exhibits excellent size-controlled capability and stability, which are conducive to ensuring the consistency of the CDs’ optical properties. The statistical analysis of 300 nanoparticles showed that the average diameter was 6.6 nm (Figure 1C), which confirmed that the preparation method had excellent size uniformity.

The optical properties of CDs were evaluated using a fluorescence spectrophotometer, and a strong fluorescence emission peak at 515 nm was observed upon excitation at 420 nm (Figure 1D). These results confirm that the green fluorescence emitted by CDs upon blue light excitation is suitable for biological fluorescence tracking, and their high fluorescence intensity and stability are sufficient to satisfy the requirements of subsequent experiments. In addition, UV-Vis spectroscopy showed that CDs had characteristic absorption in the ultraviolet-visible region. The specific analysis will be discussed in Section 3.2 with the absorption results of ZIF-8 and ZIF-8@CDs.

In general, through transmission electron microscopy observation and a series of optical performance tests, we obtained detailed data on the microstructure and optical properties of CDs. These results strongly prove that the CDs prepared by hydrothermal method with methanol as the medium not only have ideal monodispersity and size consistency in structure, but also show significant advantages in optical properties, especially in terms of fluorescence intensity and stability, making them an ideal candidate material in this paper.

### 3.2. Morphological Physicochemical Characterization of ZIF-8@CDs

**Figure 2:**
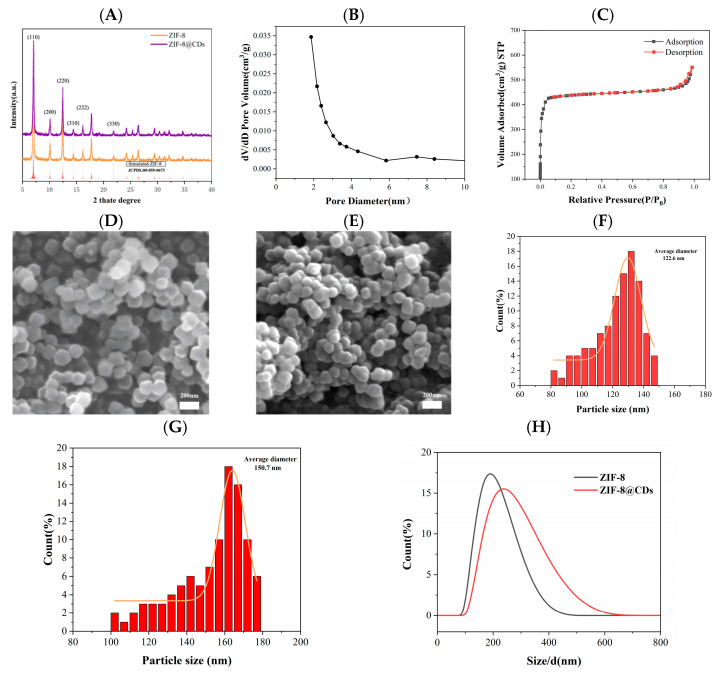
(**A**) XRD patterns of ZIF-8 and ZIF-8@CDs nanoparticles; (**B**) Pore size distribution curve of ZIF-8@CDs; (**C**) Nitrogen adsorption–desorption isotherm of ZIF-8@CDs; (**D**) SEM image of ZIF-8. Scale bar: 200 nm; (**E**) SEM image of ZIF-8@CDs. Scale bar: 200 nm; (**F**) Particle size distribution of ZIF-8; (**G**) Particle size distribution of ZIF-8@CDs; (**H**) Particle size distribution diagram of ZIF-8 and ZIF-8@CDs measured by DLS.

To verify the crystal structure and purity of the synthesized product, XRD analysis was performed on the hydrothermally synthesized ZIF-8@CDs nanoparticles (Figure 2A). The crystal structure of ZIF-8 was confirmed by XRD, and its standard crystallographic data were referenced as follows: cubic crystal system, space group I43m (No. 217), lattice parameters a = b = c = 17.0 Å, α = β = γ = 90°, and SOD-type zeolite topology. The XRD pattern of as-synthesized ZIF-8@CDs exhibits distinct characteristic diffraction peaks at 2θ ≈ 7.3° (110), 10.4° (200), 12.8° (220), 14.7° (310), 16.4° (222), and 22.1° (330), which are in full agreement with the standard ZIF-8 peaks (PDF card No. 00-059-0673)—confirming the successful synthesis of ZIF-8@CDs with high crystallinity and intact SOD topology. For the ZIF-8@CDs composite, its XRD pattern retains all the characteristic peaks of ZIF-8, indicating that the introduction of CDs does not destroy the crystal structure of ZIF-8. The slight decrease in peak intensity of ZIF-8@CDs may be attributed to the uniform loading of CDs on the surface of ZIF-8, which is a common phenomenon in MOF-based composite materials.

BET specific surface area analysis was performed on the synthesized nanomaterials (NPs), and the results revealed that ZIF-8 exhibited a specific surface area of 832 m^2^/g, a pore volume of 0.87 cm^3^/g, and a pore diameter of 0.48 nm (Table 1). Its pore size matched the dimensions of doxorubicin and siRNA, meeting the requirements for gene drug carriers. After the loading of CDs, the specific surface area of ZIF-8@CDs reduced to 644 m^2^/g, and the pore volume reduced to 0.74 cm^3^/g (Figure 2B), while the pore size was maintained at 0.48 nm, indicating that CDs successfully occupied the surface and inner walls of ZIF-8 without changing its primary pore structure. The adsorption isotherm shows the hysteresis loop and the later saturation platform (Figure 2C), reflecting that the material has uniform pore size distribution and capillary condensation characteristics. The composite carrier exhibits a considerable specific surface area and retains stable pore size, ensuring efficient loading and controlled release of macromolecules such as siRNA, thus confirming the feasibility of the CDs modification design for functional modification while maintaining the structural integrity of ZIF-8 pores.

The morphology and structure of the nanoparticles were characterized via scanning electron microscopy (SEM). The results demonstrated that the nanoparticles exhibited a regular polyhedral morphology with good dispersibility (Figure 2D,E). No aggregation was observed, which further confirmed that the preparation process of the composite material did not destroy the crystalline structure of ZIF-8. The surface of ZIF-8@CDs appeared slightly rough but still retained the hexagonal periodic structure, indicating that CDs were successfully immobilized on the particle surface and within the pores. This slight surface roughness was attributed to the tight binding between CDs and ZIF-8. The preservation of the regular polyhedral morphology and intrinsic porosity of the composite material further verified that the proposed synthetic strategy effectively retained the structural advantages of ZIF-8 while endowing it with CD-based functionalities. For size statistics of the nanoparticles in the SEM images, when the count reached 100 nanoparticles, the average particle size of ZIF-8 was approximately 122 nm (Figure 2F), and that of ZIF-8@CDs was approximately 150 nm (Figure 2G).

The particle size distribution and Zeta potential of the NPs were determined using the dynamic light scattering (DLS) technique and Zeta potential measurement. The experimental results are summarized in (Table 2). Through this analysis, the particle size distribution and surface charge characteristics of ZIF-8, CDs, and ZIF-8@CDs composites were clarified, and the correlation between their surface chemical properties and biocompatibility was explored.

Zeta potential measurements revealed that the surface charge of ZIF-8 was +36 mV. After loading the CDs, the surface potential of ZIF-8@CDs decreased to 25.7 mV, still maintaining a positive charge. The presence of abundant -OH/-COOH functional groups rendered the surface of CDs electrically negative. Since the particles were too small for rapid deposition, accurate quantification could not be achieved. This moderately positive charge was conducive to interacting with negatively charged cell membranes to promote endocytosis.

DLS measurement (Figure 2H) showed that the hydrated particle size of ZIF-8 nanoparticles was about 180 nm, which was larger than that of TEM. The reason was that the positively charged surface caused the thickening of the hydration layer, and the size of the ZIF-8@CDs nanoparticles increased to 205 nm, confirming that CDs were successfully loaded on the surface and pores of ZIF-8. Although the particle size of the composite particles increased, it was still in the range of 200 nm, which was in line with the optimal size range of nano-drug carriers to promote cell phagocytosis and facilitate efficient intracellular delivery. The CDs loaded on the surface of ZIF-8@CDs, as well as the ZIF-8 themselves, are rich in hydrophilic functional groups such as carboxyl and hydroxyl groups. These groups can easily form hydrogen bonds with water molecules in aqueous solution and adsorb a large amount of solvent molecules, creating a considerable hydration layer. This results in DLS measurements being larger than TEM results, but this size difference is a common and reasonable phenomenon in nanoparticle characterization.

**Figure 3:**
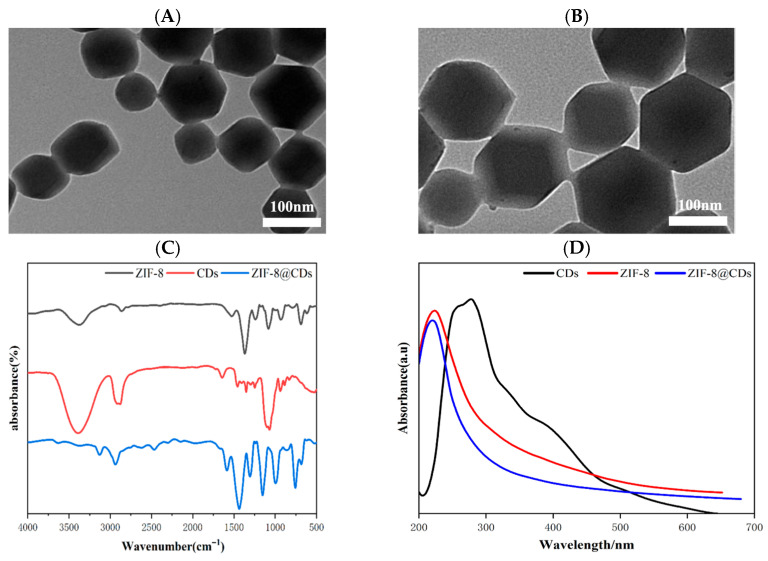
(**A**) TEM diagram of ZIF-8. Scale bar: 100 nm; (**B**) TEM diagram of ZIF-8@CDs. Scale bar: 100 nm; (**C**) FTIR of CDs, ZIF-8 and ZIF-8@CDs; (**D**) UV-Vis absorption spectra of CDs, ZIF-8 and ZIF-8@CDs.

TEM was used to characterize ZIF-8 and ZIF-8@CDs. The results indicate that the unmodified ZIF-8 exhibits a clear hexagonal close-packed structure, with good particle dispersibility, uniform particle size, and diameters of approximately 80–120 nm (Figure 3A). After the dense adsorption of CDs on the surface and near-surface regions of ZIF-8, the particle size increases to 100–180 nm (Figure 3B) ZIF-8@CDs still retain ZIF-8′s typical well-defined polyhedral morphology, which is a characteristic feature of crystalline ZIF-8 (amorphous or poorly crystalline ZIF materials generally appear as irregular, blurry aggregates). Together with XRD results (with characteristic diffraction peaks matching the standard ZIF-8 card), this confirms that ZIF-8@CDs possess good crystallinity. The polyhedral framework edges of both ZIF-8 and ZIF-8@CDs are clear and well-defined, without noticeable structural collapse, fragmentation, or amorphous regions. Moreover, the surface-loaded CDs are evenly distributed and do not compromise the main framework or morphology of ZIF-8, indicating that ZIF-8@CDs maintain excellent structural integrity after composite modification.

FTIR characterization showed that (Figure 3C): The characteristic peaks of ZIF-8 nanoparticles at 3135 cm^−1^ and 2924 cm^−1^ correspond to the stretching vibrations of C-H bonds in the 2-methylimidazole ligand. The absorption peaks at 1582 cm^−1^, 1485 cm^−1^, and 1384 cm^−1^ are attributed to the skeletal vibrations of the imidazole ring. The characteristic peak around 600 cm^−1^ is related to the Zn-N coordination bond formed between Zn^2+^ in the ZIF-8 framework and the N atoms of the ligand. The infrared spectrum of CDs showed that the strong absorption peak at 1634 cm^−1^ was the stretching vibration characteristic peak of the C = O bond in the surface carboxyl (-COOH), which proved that the surface of CDs was rich in hydrophilic carboxyl functional groups [20]. The broad peak near 3420 cm^−1^ corresponds to the stretching vibration of hydroxyl (-OH) on the surface of CDs. It is worth noting that compared with CDs, the C = O stretching vibration peak originally located at 1634 cm^−1^ in ZIF-8@CDs is significantly blue-shifted to 1700 cm^−1^. The main reason for the displacement is the interfacial interaction between CDs and ZIF-8: the C = O in the carboxyl group on the surface of CDs can not only form a coordination bond with Zn^2+^ on the ZIF-8 skeleton but also form a hydrogen bond with the amino group (-NH) in the 2-methylimidazole ligand [26]. Such interactions reduce the electron cloud density of the C = O bond and eventually lead to a blue shift in the characteristic peak [14]. This result confirms that CDs and ZIF-8 are not simply physically mixed but are successfully compounded through surface functional group interactions. The characteristic peak at 600 cm^−1^ in the spectrum corresponds to the Zn-N coordination bond absorption of the ZIF-8 framework, rather than a characteristic peak of ZnO impurities. According to the literature, the characteristic infrared absorption peak of ZnO is mainly in the 400–500 cm^−1^ range (with a typical peak around 437 cm^−1^), corresponding to the stretching vibration of the Zn-O bond [36]. None of the spectra of our samples showed this characteristic peak, ruling out the possibility of ZnO impurity formation during the synthesis. In contrast, the stretching vibration peak of the Zn-N coordination bond in ZIF-8, due to coordination between Zn^2+^ and the N atoms of 2-methylimidazole ligands, generally appears in the 500–650 cm^−1^ range [50], which exactly matches the peak at 600 cm^−1^ observed in our experiment. Moreover, in the spectrum of the ZIF-8@CDs composite, not only is the Zn-N characteristic peak at 600 cm^−1^ retained, but the skeletal vibration peaks of the imidazole ring in ZIF-8 are also fully preserved, with only a slight decrease in peak intensity. This further confirms that the introduction of CDs does not disrupt the main framework structure of ZIF-8. These results indicate that CDs successfully combine with ZIF-8 through surface functional group interactions, and the crystalline framework structure of ZIF-8 remains intact after the composite formation, providing a structural foundation for the subsequent study of the material’s stability and functional properties.

UV-Vis analysis (Figure 3D) showed that CDs (black line) exhibit a characteristic peak at 282 nm corresponding to the π-π* transition of C = C, and the shoulder peak at 400 nm is attributed to the n→π* electronic transition of oxygen-containing functional groups on the CD surface (such as -COOH and -OH). ZIF-8 (red line) and ZIF-8@CDs (blue line) display an absorption trend that first increases and then decreases within the 200–700 nm range. The blue shift in the characteristic peak at 230 nm in ZIF-8@CDs is attributed to energy transfer or electron coupling between CDs and ZIF-8, confirming the effective combination of the two and the generation of synergistic optical properties.

### 3.3. Detection of Fenton-like Catalytic Activity of ZIF-8@CDs

**Figure 4:**
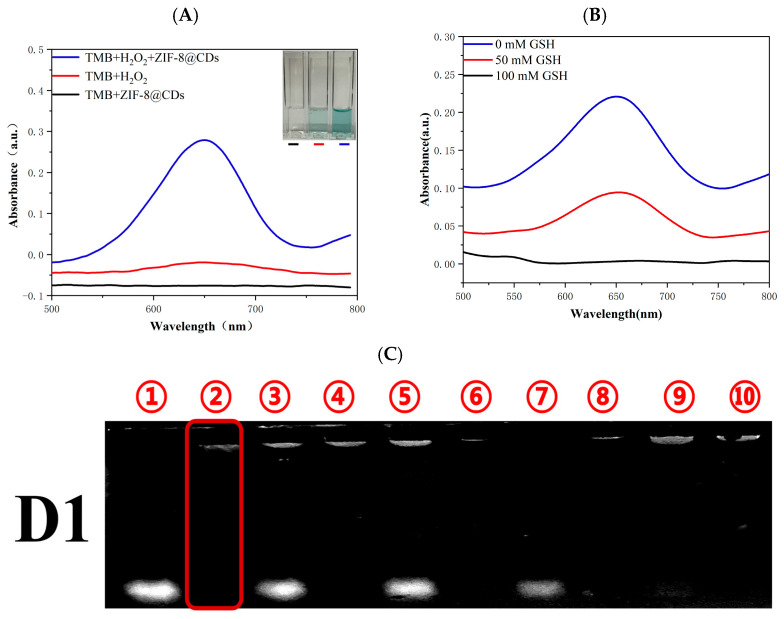
(**A**) UV-Vis absorption spectra of reaction systems with different reagents: TMB + ZIF-8@CDs (black line), TMB + H_2_O_2_ (red line), and TMB+ H_2_O_2_+ ZIF-8@CDs (blue line). Illustration: Photograph of the corresponding sample; (**B**) UV-Vis absorption curves and photographs of 0.1 mg/mL ZIF-8@CDs with 10 mM TMB and H_2_O_2_ at pH 4.0 in the presence of different concentrations of GSH. Incubate the mixture at 50 °C for 30 min; (**C**) Gel retardation profile of herring sperm DNA incubated with ZIF-8@CDs composites at varied DNA concentrations. Lanes: ① (control): 100 ng/μL herring sperm DNA; ②: 100 ng/μL herring sperm DNA + ZIF-8@CDs; ③ (control): 80 ng/μL herring sperm DNA; ④: 80 ng/μL herring sperm DNA + ZIF-8@CDs; ⑤ (control): 60 ng/μL herring sperm DNA; ⑥: 60 ng/μL herring sperm DNA + ZIF-8@CDs; ⑦ (control): 30 ng/μL herring sperm DNA; ⑧: 30 ng/μL herring sperm DNA + ZIF-8@CDs; ⑨ (control): 10 ng/μL herring sperm DNA; ➉: 10 ng/μL herring sperm DNA + ZIF-8@CDs.

The Fenton-like catalytic activity of ZIF-8@CDs composites was evaluated using TMB as the probe. The results are shown in (Figure 4A). The results confirmed that ZIF-8@CDs exhibit remarkable Fenton-like catalytic activity: via the TMB colorimetric assay, it was observed that they catalyze H_2_O_2_ to generate ·OH, as indicated by the absorbance of blue oxidized TMB (ox-TMB) at 652 nm increases linearly with the catalyst concentration. Furthermore, they can induce ROS bursts in the tumor microenvironment. The GSH detection system established simultaneously showed, as illustrated in (Figure 4B), that ox-TMB was reduced to a colorless state by GSH; within the concentration range of 0–100 μM, the absorbance declined in a dose-dependent fashion as the GSH concentration increased. This proved that the carrier could simultaneously consume GSH in tumor cells and break the oxidation-antioxidation balance. The above characteristics make ZIF-8@CDs have the integrated function of catalytic therapy and diagnosis and treatment: producing reactive oxygen species through pH/H_2_O_2_ responsiveness, synergistically consuming GSH to enhance oxidative damage of tumor cells, and providing support for chemotherapy-gene-catalytic multi-mode synergistic therapy.

### 3.4. Study on the Adsorption of DNA by ZIF-8@CDs

The adsorption performance was evaluated by 20 bp herring sperm DNA simulated siRNA: Gel retardation experiments showed that the DNA adsorption capacity of ZIF-8@CDs gradually increased with the increase in DNA content (10–100 ng). When DNA reached 100 ng, channel 2 still did not show a band, indicating that ZIF-8@CDs had strong loading and protection ability for DNA (Figure 4C), confirming ZIF-8@CDs high loading and protection ability. Quantitative determination of the amount of DNA bound to the complex by centrifugation: When the DNA concentration reached 100 μg/mL, the adsorption efficiency decreased (Table 3), reflecting that the carrier load tended to be saturated. The experimental results systematically verified the adsorption capacity and saturation characteristics of ZIF-8@CDs as a nucleic acid drug carrier, which provided a key experimental basis for optimizing siRNA loading conditions and evaluating carrier delivery efficiency, and supported the precise design and performance improvement of subsequent targeted drug delivery systems.

### 3.5. Assessment of Cellular Uptake

**Figure 5:**
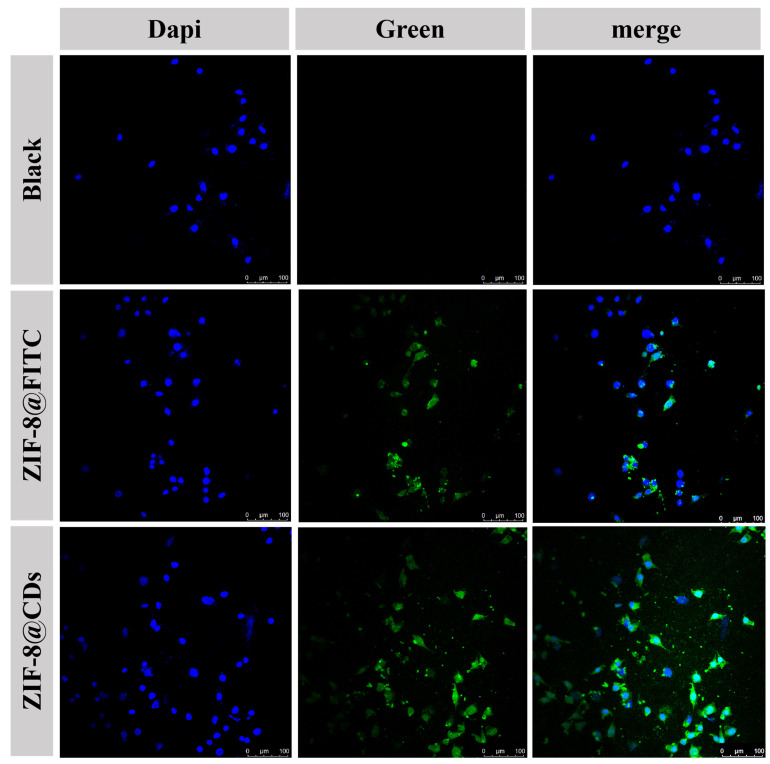
CLSM of HepG2 cells treated with the blank control group, ZIF-8@FITC group, and ZIF-8@CDs group after DAPI staining. Scale bar: 200 nm.

The cellular uptake efficiency of the nanocarrier was verified via FITC-labeling tracing: confocal microscopy (Figure 5) showed that ZIF-8@CDs and ZIF-8@FITC groups showed significant green fluorescence in the cytoplasm and endosome regions after co-culture for 4 h, confirming their efficient endocytosis. The fluorescence intensity and stability of the ZIF-8@CDs group were better than those of ZIF-8@FITC, which was attributed to the inherent strong anti-quenching fluorescence characteristics of CDs. The co-localization analysis of DAPI nuclear staining and nanoparticle fluorescence showed that the carrier did not interfere with the normal physiological activities of cells, and the persistent fluorescence characteristics of CDs can track the intracellular delivery and release behavior of drugs/genes throughout the process, providing a reliable technical means for visual monitoring of carrier-cell interactions.

### 3.6. Detection of Intracellular ROS

**Figure 6:**
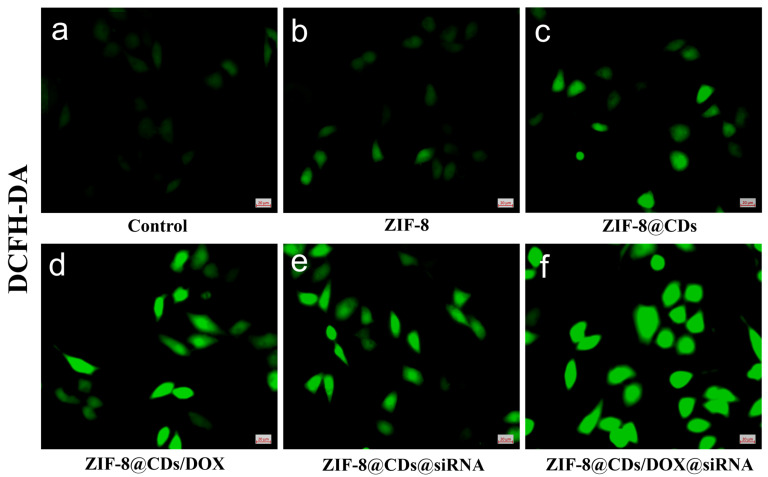
CLSM of ROS generation in HepG2 cells based on DCFH-DA fluorescence intensity under the following conditions: blank control (**a**), ZIF-8 (**b**), ZIF-8@CDs (**c**), ZIF-8@CDs/DOX (**d**), ZIF-8@CDs@siRNA (**e**), and ZIF-8@CDs/DOX@siRNA (**f**). Scale bar: 20 μm.

The DCFH-DA fluorescence probe assay confirmed that the green fluorescence intensity in the ZIF-8@CDs treatment group (Figure 6c) was significantly higher than that in the control group (Figure 6a) and the ZIF-8 group (Figure 6b). This finding indicates that the carrier induced an intracellular ROS surge via Fenton-like catalysis, thereby causing oxidative stress damage to cells. The DOX-loaded ZIF-8@CDs group (Figure 6d) further aggravated ROS accumulation and lipid peroxidation, which was consistent with the literature reports that DOX promotes ROS production. The ZIF-8@CDs/DOX@siRNA triple composite system (Figure 6f) significantly increased the apoptosis rate of cancer cells compared with that in the single drug treatment group, confirming that the carrier exhibited multiple functions: efficiently delivering DOX/siRNA to target cells, catalyzing the production of ROS to enhance oxidative damage, synergistically silencing genes to reverse drug resistance, and enhancing anti-tumor effects through multi-channel synergistic mechanisms.

### 3.7. Cytotoxicity Detection

**Figure 7:**
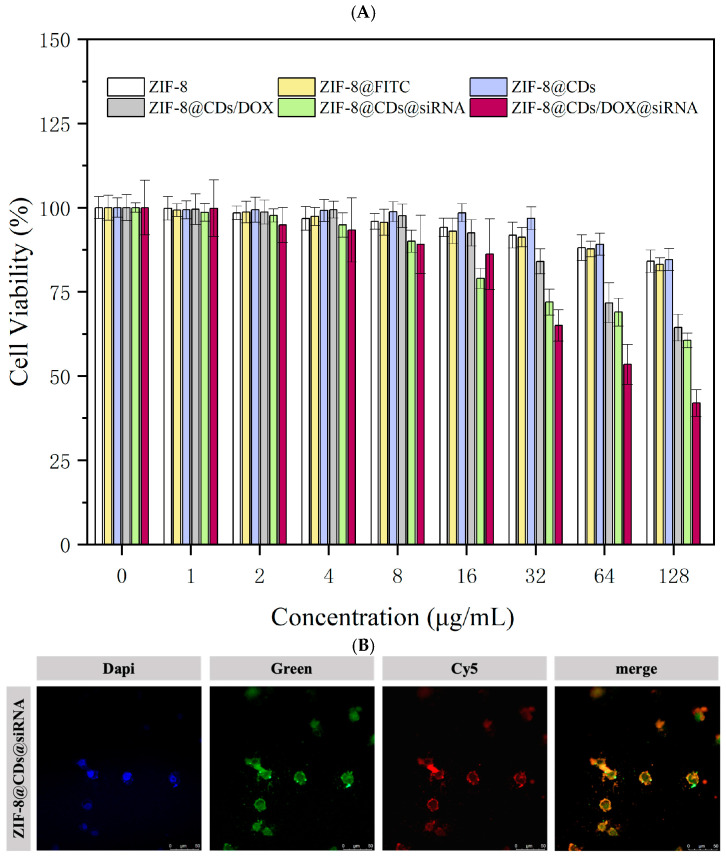
(**A**) Cytotoxicity testing of HepG2 cells after exposure to ZIF-8, ZIF-8@FITC, ZIF-8@CDs, ZIF-8@CDs/DOX, ZIF-8@CDs@siRNA, and ZIF-8@CDs/DOX@siRNA at concentrations of 0, 1, 2, 4, 8, 16, 32, 64, and 128 μg/mL. Error bars represent standard deviation from the mean (*n* = 3); (**B**) After treatment with DAPI and Cy5, and taking advantage of the intrinsic fluorescence properties of ZIF-8@CDs@siRNA, the results of the cellular uptake experiment were examined using CLSM. Scale bar: 50 µm.

The MTT assay (Figure 7A) showed that the cell viability of the untreated group was nearly 100%, which confirmed the reliability of the experimental system; the cell viability of cells treated with blank carriers (ZIF-8 and ZIF-8@CDs) remained ≥85% even when exposed to the highest carrier concentration for 12 h, indicating that the material itself has low toxicity and excellent biocompatibility. In the ZIF-8@CDs/DOX@siRNA group, the cell viability decreased to 45%, which was attributed to the release of DOX and siRNA triggered by the intracellular acidic environment; meanwhile, its inhibitory effect on cell viability enhanced with increasing carrier concentration. These results confirmed that the composite carrier significantly suppresses the viability of HepG2 cells only when it is loaded with therapeutic agents, highlighting that it has favorable therapeutic potential and safety as a targeted delivery system.

### 3.8. Cell Uptake Experiment of ZIF-8@CDs Carrying siRNA

Given the inherent characteristics of siRNA—namely, its negative charge and poor cellular membrane permeability—the ZIF-8@CDs composite carrier was employed for efficient siRNA delivery. Confocal imaging of Cy5-labeled siRNA (Figure 7B) revealed that the red fluorescence signal (from Cy5-siRNA) and the green fluorescence signal (from the CDs component of the carrier) were highly co-localized within cells. This finding confirmed that siRNA had been successfully loaded onto the carrier and was co-endocytosed with the carrier into cellular endosomes, thereby overcoming the key limitations of conventional siRNA transfection methods.

### 3.9. Cell Apoptosis Assay

**Figure 8:**
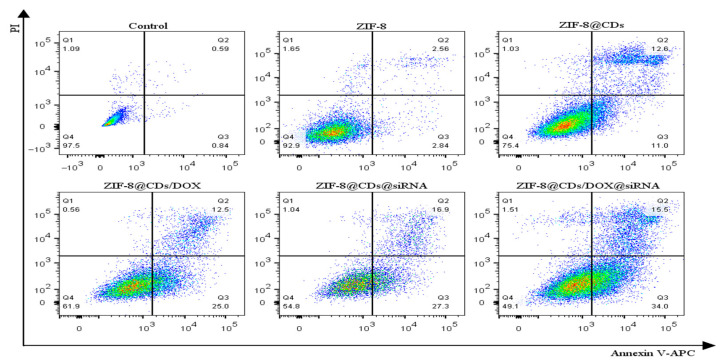
Annexin V-APC apoptosis staining and propidium iodide flow cytometry showed the apoptosis of Control, ZIF-8, ZIF-8@CDs, ZIF-8@CDs/DOX, ZIF-8@CDs@siRNA, and ZIF-8@CDs/DOX@siRNA groups.

Cell apoptosis was assessed via flow cytometry. The results (Figure 8) demonstrated that the proportion of viable cells in the blank group was 97.5%, corresponding to a low apoptosis rate. For the ZIF-8 group, the viable cell proportion was 92.9% (apoptosis rate: 5.4%); for the ZIF-8@CDs group, this value decreased to 75.4% (apoptosis rate: 23.6%); and for the ZIF-8@CDs/DOX group, the viable cell proportion was 61.9% (apoptosis rate: 37.5%). These findings confirmed that the intracellular release of DOX enhanced the tumor cell-killing efficacy. The ZIF-8@CDs/siRNA group exhibited a viable cell proportion of 54.8% (apoptosis rate: 44.2%), verifying the successful transfection of siRNA and its induction of gene-mediated apoptosis. Notably, the dual-drug-loaded ZIF-8@CDs/DOX@siRNA group showed a viable cell proportion of 49.1% (apoptosis rate: 49.5%). Although this apoptosis rate was not significantly higher than those of the single-drug groups (DOX-only group: 39.5%; siRNA-only group: 44.2%), the results still indicated a tendency toward synergistic therapeutic effects. Collectively, these data systematically validated that the ZIF-8@CDs carrier can efficiently deliver DOX and siRNA to induce tumor cell apoptosis, wherein the CDs component contributes additional pro-apoptotic effects through its photosensitive properties.

## 4. Discussion

In summary, the researchers developed an effective strategy for immobilizing CDs on the surface and near-surface of ZIF-8 nanoparticles, thereby constructing a MOF composite. This composite was successfully applied for the co-delivery of the anticancer drug DOX and siRNA. Characterization results demonstrated that the ZIF-8@CDs nanocomposite exhibited an average particle size of 205 ± 13 nm, a surface zeta potential of 25.7 mV, a specific surface area of 644 m^2^/g, and a maintained pore size of 0.48 nm. This uniform, positively charged nanostructure, combined with its favorable pore characteristics, endowed the material with excellent fluorescence properties and suitability as a gene/drug carrier. Furthermore, the material displayed extremely low toxicity and outstanding biocompatibility, laying a robust structural foundation for its conjugation with DOX and siRNA. Notably, this carrier exhibited significant intrinsic Fenton-like catalytic activity. Experiments involving TMB colorimetry, GSH consumption, and intracellular ROS burst collectively confirmed that it could induce hepatocellular carcinoma cell apoptosis via catalytic generation of ROS, enabling chemodynamic therapy. By co-loading DOX and siRNA targeting the Survivin gene, the carrier efficiently delivered therapeutic components into HepG2 cells and disintegrated in the acidic tumor microenvironment to release DOX and siRNA. The synergistic treatment group showed a markedly enhanced tumor cell-killing effect, with a cell apoptosis rate of 49.5%, verifying the multi-modal synergistic mechanism integrating chemotherapy, gene silencing, and catalytic therapy. CLSM studies revealed that the ZIF-8@CDs/DOX@siRNA nanosystem exhibited high contrast in intracellular fluorescence imaging, indicating its potential for bioimaging applications and facilitating the intracellular localization of anticancer drugs. We anticipate that the design of this pH-responsive multi-modal nanocarrier system will provide theoretical and experimental foundations for the development of multifunctional nanodrug carriers and the advancement of hepatocellular carcinoma treatment strategies.

## Figures and Tables

**Table 1:** Specific surface area, pore volume pore size parameters of Nano carrier NPs.

NPs	S_BET_ (m^2^/g)	V_P_ (cm^3^/g)	Pore Size (nm)
ZIF-8	832	0.87	0.48
ZIF-8@CDs	644	0.74	0.48

**Table 2:** Loat Granules NPs Power and particle size parameters.

NPs	Zeta Potential (mV)	Size (nm)
ZIF-8	36	180 ± 5
CDs	-	6.6 ± 1.1
ZIF-8@CDs	25.7	205 ± 13

**Table 3:** Adsorption experiments of different NPs on 20 bp DNA.

NPs Materials	Actual Initial DNA Concentration (μg/mL)	DNA Adsorption Capacity (μg/mg)	Adsorption Efficiency (%)
ZIF-8@CDs	100	36.25	72.5
	80	32.05	80.1
	60	25.35	84.5
	30	10.55	70.3
	10	3.10	62.1

## Data Availability

The data and materials generated and analyzed during this paper can be obtained from the corresponding author upon reasonable request.

## References

[1] Deng Y., Tan J., Zu J., Xing Z., Yang Z., Zhang Y., Bo Y., Gao X., Xie E., Wang Y. (2025). Trends and health inequalities of hepatitis virus-associated liver cancer mortality during 1990-2050. Ann. Hepatol..

[2] Pei Z., Chen S., Ding L., Liu J., Cui X., Li F., Qiu F. (2022). Current perspectives and trend of nanomedicine in cancer: A review and bibliometric analysis. J. Control. Release.

[3] Reshmi R., Jiju K.R., Suma S., Nair A.S. (2023). Folic acid grafted aminated zeolitic imidazolate framework (ZIF-8) as pH responsive drug carrier for targeted delivery of curcumin. J. Drug Deliv. Sci. Technol..

[4] Sulaiman C., George B.P., Balachandran I., Abrahamse H. (2022). Photoactive Herbal Compounds: A Green Approach to Photodynamic Therapy. Molecules.

[5] Chen B., Wu S., Ye Q. (2021). Fabrication and characterization of biodegradable KH560 crosslinked chitin hydrogels with high toughness and good biocompatibility. Carbohydr. Polym..

[6] Mi P., Cabral H., Kataoka K. (2020). Ligand-Installed Nanocarriers: Ligand-Installed Nanocarriers toward Precision Therapy (Adv. Mater. 13/2020). Adv. Mater..

[7] Ohkoshi-Yamada M., Kamimura K., Shibata O., Morita S., Terai S. (2020). Efficacy and Safety of the Radiotherapy for Liver Cancer: Assessment of Local Controllability and Its Role in Multidisciplinary Therapy. Cancers.

[8] Cai X., Bao X., Wu Y. (2022). Metal–Organic Frameworks as Intelligent Drug Nanocarriers for Cancer Therapy. Pharmaceutics.

[9] An J., Hu Y.G., Cheng K., Li C., Zhang M.Z. (2020). ROS-augmented and tumor-microenvironment responsive biodegradable nanoplatform for enhancing chemo-sonodynamic therapy. Biomaterials.

[10] Zong Z., Tian G., Wang J., Fan C., Yang F., Guo F. (2022). Recent Advances in Metal–Organic-Framework-Based Nanocarriers for Controllable Drug Delivery and Release. Pharmaceutics.

[11] Jiang S., Cai G., Yang Z., Shi H., Zeng H., Ye Q., Hu Z., Wang Z. (2024). Biomimetic Nanovesicles as a Dual Gene Delivery System for the Synergistic Gene Therapy of Alzheimer’s Disease. ACS Nano.

[12] Su J., Chen X., Ye F., Liu C., Liang J., Zhang X., Guo X. (2024). Celastrol Derivative/DOX Co-Assembled Nanodrug for Enhanced Antitumor Therapy. Curr. Drug Deliv..

[13] Wang N., Cheng X., Li N., Wang H., Chen H. (2019). Nanocarriers and Their Loading Strategies. Adv. Healthc. Mater..

[14] Wang J., Fu Y., Gu Z., Pan H., Zhou P., Gan Q., Yuan Y., Liu C. (2024). Multifunctional Carbon Dots for Biomedical Applications: Diagnosis, Therapy, and Theranostic. Small.

[15] Deb A., Chowdhury D. (2024). Biogenic Carbon Quantum Dots: Synthesis and Applications. Curr. Med. Chem..

[16] Zhu P., Liu Y., Tang Y., Zhu S., Liu X., Yin L., Liu Q., Yu Z., Xu Q., Luo D. (2024). Bi-doped carbon quantum dots functionalized liposomes with fluorescence visualization imaging for tumor diagnosis and treatment. Chin. Chem. Lett..

[17] Li W., Wang X., Lin J., Meng X., Wang L., Wang M., Jing Q., Song Y., Vomiero A., Zhao H. (2024). Controllable and large-scale synthesis of carbon quantum dots for efficient solid-state optical devices. Nano Energy.

[18] Peng J., Gong P., Li S., Kong F., You J. (2019). A smart bioresponsive nanosystem with dual-modal imaging for drug visual loading and targeted delivery. Chem. Eng. J..

[19] Wu B., Li K., Sun F., Niu J., Zhu R., Qian Y., Wang S. (2021). Trifunctional Graphene Quantum Dot@LDH Integrated Nanoprobes for Visualization Therapy of Gastric Cancer (Adv. Healthcare Mater. 16/2021). Adv. Healthc. Mater..

[20] Mai X.-D., Thi Kim Chi T., Nguyen T.-C., Ta V.-T. (2020). Scalable synthesis of highly photoluminescence carbon quantum dots. Mater. Lett..

[21] Yin X., Ran S., Cheng H., Zhang M., Sun W., Wan Y., Shao C., Zhu Z. (2022). Polydopamine-modified ZIF-8 nanoparticles as a drug carrier for combined chemo-photothermal osteosarcoma therapy. Colloids Surf. B Biointerfaces.

[22] Wang L., Tong L., Xiong Z., Chen Y., Zhang P., Gao Y., Liu J., Yang L., Huang C., Ye G. (2024). Ferroptosis-inducing nanomedicine and targeted short peptide for synergistic treatment of hepatocellular carcinoma. J. Nanobiotechnol..

[23] Zhao J., Zhang C., Wang W., Li C., Mu X., Hu K. (2022). Current progress of nanomedicine for prostate cancer diagnosis and treatment. Biomed. Pharmacother..

[24] Wang Z., Yan L., Song Z., Zhang J., Yang Y., Liu X. (2025). Revealing the in-situ growth mechanism of carbon dots confined in ZIF-8 as multicolor fluorescent material with high photothermal stability. J. Colloid Interface Sci..

[25] Ti Y., Qin Z., Guo X., Wu Z., Jiang Q., Dong B., Fawole O.A., Wu D., Chen S., Li X. (2025). Fabrication of Dual Photodynamic Enhanced Antimicrobial CDs@ZIF-8/Polycaprolactone/Ethyl Cellulose Nanofibrous Films for Fruit Preservation. Adv. Sci..

[26] Li H.-J., Wang H., Si T., Wang H., Huang S., Wu Y., Liao Q., Wang D., Li Y. (2024). Tailoring Morphology and Fluorescence Properties of Zeolitic Imidazolate Frameworks via Carbon Dots. ACS Appl. Nano Mater..

[27] Tian D., Yang S., Liu Y., Zhou H., Zhou P., Xiong Z., Yao G., Lai B. (2022). Zeolite imidazole framework-8-derived nitrogen-doped nanocarbon boosted Fenton-like oxidation: Another sustainable path for Fe(III)/Fe(II) circulation. Environ. Funct. Mater..

[28] Yang W., Hong P., Yang D., Yang Y., Wu Z., Xie C., He J., Zhang K., Kong L., Liu J. (2021). Enhanced Fenton-like degradation of sulfadiazine by single atom iron materials fixed on nitrogen-doped porous carbon. J. Colloid Interface Sci..

[29] Lian T., Xu L., Piankova D., Yang J.-L., Tarakina N.V., Wang Y., Antonietti M. (2024). Metal-organic framework derived crystalline nanocarbon for Fenton-like reaction. Nat. Commun..

[30] Wang Z., Zhang M., Wang J., Kakavandi B., Niu J., Li W.-W., Bao Y. (2025). ZIF-Derived Catalyst with Co–Co/Co–N Dual Active Sites for Boosting Mixed Pathway Decontamination in Fenton-like Catalysis. Environ. Sci. Technol..

[31] Yu S.-S., Gu B., Wei T.-T., Shu X.-X., Liu L.-L., Huang M.-J., Chen J.-J., Yu H.-Q. (2025). Tailoring Interfacial Active Sites and Valence States via Ligand Modulation in ZIF-Derived Catalysts for Enhanced Fenton-like Reactions. ACS Appl. Mater. Interfaces.

[32] Nguyen N.T., Kim J., Le X.T., Lee W.T., Lee E.S., Oh K.T., Choi H.-G., Youn Y.S. (2023). Amplified Fenton-Based Oxidative Stress Utilizing Ultraviolet Upconversion Luminescence-Fueled Nanoreactors for Apoptosis-Strengthened Ferroptosis Anticancer Therapy. ACS Nano.

[33] Zhao C., Liu Z., Chang C.-C., Chen Y.-C., Zhang Q., Zhang X.-D., Andreou C., Pang J., Liu Z.-X., Wang D.-Y. (2023). Near-Infrared Phototheranostic Iron Pyrite Nanocrystals Simultaneously Induce Dual Cell Death Pathways via Enhanced Fenton Reactions in Triple-Negative Breast Cancer. ACS Nano.

[34] Wu A., Han M., Ni Z., Li H., Chen Y., Yang Z., Feng Y., He Z., Zhen H., Wang X. (2024). Multifunctional Sr/Se co-doped ZIF-8 nanozyme for chemo/chemodynamic synergistic tumor therapy via apoptosis and ferroptosis. Theranostics.

[35] Zhong Y., Peng Z., Peng Y., Li B., Pan Y., Ouyang Q., Sakiyama H., Muddassir M., Liu J. (2023). Construction of Fe-doped ZIF-8/DOX nanocomposites for ferroptosis strategy in the treatment of breast cancer. J. Mater. Chem. B.

[36] Babayevska N., Przysiecka Ł., Iatsunskyi I., Nowaczyk G., Jarek M., Janiszewska E., Jurga S. (2022). ZnO size and shape effect on antibacterial activity and cytotoxicity profile. Sci. Rep..

[37] Dai L., Ma W., Song Z., Lu B., He Y., Zhang J., Wei D., Wang B., Li G., Gao D. (2025). Targeted and Synergistic Codelivery of Chemotherapeutic and Nucleic Acid Drugs by Liposome-Coated MPDA Nanoparticles for Advanced Prostate Cancer Treatment. ACS Appl. Mater. Interfaces.

[38] Wang Q., Du Y., Zheng J., Shi L., Li T. (2024). G-Quadruplex-Programmed Versatile Nanorobot Combined with Chemotherapy and Gene Therapy for Synergistic Targeted Therapy. Small.

[39] Huang L., Guo W., Zhao T., Feng Y., Li Y., An Q., Li C., Tian Y., Zhang H., Zhou C. (2025). Microfluidic fabrication of lipid nanoparticles for co-delivery of siRNA and hydroxychloroquine: An engineered theranostic platform for enhanced breast cancer treatment. Chem. Eng. J..

[40] Yuan J., Wang J., Song M., Zhao Y., Shi Y., Zhao L. (2024). Brain-targeting biomimetic disguised manganese dioxide nanoparticles via hybridization of tumor cell membrane and bacteria vesicles for synergistic chemotherapy/chemodynamic therapy of glioma. J. Colloid. Interface Sci..

[41] Li M., Li T., Liu Y., Han D., Wu S., Gong J. (2025). Dual Cascade-Responsive Multifunctional Nanoparticles to Overcome Bacterium-Induced Drug Inactivation and Enhanced Photodynamic and Chemo-Immunotherapy of Pancreatic Cancer. Small.

[42] Zheng X., Zhong T., Zhao H., Huang F., Huang W., Hu L., Xia D., Tian S., Shu D., He C. (2024). MnO(2)-based capacitive system enhances ozone inactivation of bacteria by disrupting cell membrane. Water Res..

[43] Wu W., Yu X., Sun J., Han Y., Ma Y., Zhang G., Ma Q., Li Q., Xiang H. (2023). Zeolitic Imidazolate Framework (ZIF-8) Decorated Iron Oxide Nanoparticles Loaded Doxorubicin Hydrochloride for Osteosarcoma Treatment—In vitro and in vivo Preclinical Studies. Int. J. Nanomed..

[44] Mi X., Lou Y., Wang Y., Dong M., Xue H., Li S., Lu J., Chen X. (2023). Glycyrrhetinic Acid Receptor-Mediated Zeolitic Imidazolate Framework-8 Loaded Doxorubicin as a Nanotherapeutic System for Liver Cancer Treatment. Molecules.

[45] Liu M., Sun C., Jiang J., Wan L., Hu C., Wen C., Huang G., Ruan Q., Wu S., Qiao D. (2023). Aptamer-Functionalized ZIF-8 Nanomedicine for Targeted Delivery of Gefitinib and siRNA to Treat Tyrosine-Kinase-Inhibitor-Resistant Nonsmall-Cell Lung Cancer. ACS Appl. Nano Mater..

[46] Zhou J., Lin G., Fu X., Qiu S., Zhang Y., Chen X., Liu Y., Wan X., Li Z., Li Y. (2025). ZIF-8-Modified Multifunctional Hydrogel Loading siRNA and DOX for Postoperative Therapy of Maxillofacial Osteosarcoma and Bone Repair. ACS Appl. Mater. Interfaces.

[47] Hettiarachchi S.D., Kirbas Cilingir E., Maklouf H., Seven E.S., Paudyal S., Vanni S., Graham R.M., Leblanc R.M. (2021). pH and redox triggered doxorubicin release from covalently linked carbon dots conjugates. Nanoscale.

[48] Yan J., Liu C., Wu Q., Zhou J., Xu X., Zhang L., Wang D., Yang F., Zhang H. (2020). Mineralization of pH-Sensitive Doxorubicin Prodrug in ZIF-8 to Enable Targeted Delivery to Solid Tumors. Anal. Chem..

[49] Khorsandi K., Keyvani-Ghamsari S., Khatibi Shahidi F., Hosseinzadeh R., Kanwal S. (2021). A mechanistic perspective on targeting bacterial drug resistance with nanoparticles. J. Drug Target..

[50] Su L., Wu Q., Tan L., Huang Z., Fu C., Ren X., Xia N., Chen Z., Ma X., Lan X. (2019). High Biocompatible ZIF-8 Coated by ZrO2 for Chemo-microwave Thermal Tumor Synergistic Therapy. ACS Appl. Mater. Interfaces.

